# Conflict and Intimacy in Emerging Adults’ Romantic Relationships and Depressive Symptoms: The Mediating Role of Identity and Couple Satisfaction

**DOI:** 10.3390/bs14110977

**Published:** 2024-10-22

**Authors:** Federica Graziano, Sofia Mastrokoukou, Elena Cattelino, Luca Rollè, Emanuela Calandri

**Affiliations:** 1 Department of Psychology, University of Torino, 10124 Torino, Italy; l.rolle@unito.it (L.R.); emanuela.calandri@unito.it (E.C.); 2 Department of Political and Social Sciences, University of Salerno, 84084 Fisciano, Italy; smastrokoukou@unisa.it; 3 Department of Social and Human Sciences, University of Valle d’Aosta, 11100 Aosta, Italy; e.cattelino@univda.it

**Keywords:** romantic relationships, emerging adult, depression, conflict, intimacy, couple satisfaction, identity

## Abstract

Romantic relationships in emerging adulthood may be associated with depression, but the role of possible mediators of this relationship remains to be explored. The present study tested a serial mediation model where intimacy and conflict in romantic relationships were predictors of depressive symptoms through the mediation of couple satisfaction and identity satisfaction. The study was conducted on a sample of 268 Italian emerging adults, 60% female (Mage = 19, ds = 1.4), all involved in a heterosexual romantic relationship. The results showed that the association between intimacy and depressive symptoms was direct and sequentially mediated through couple satisfaction and identity satisfaction. A single mediation pathway through identity satisfaction alone also emerged. The association between conflict and depressive symptoms was direct and sequentially mediated through couple satisfaction and identity satisfaction. These findings suggest the importance of helping emerging adults develop appropriate skills in managing conflict and intimacy in romantic relationships as useful resources for identity satisfaction and depression prevention.

## 1. Introduction

The construct of emerging adulthood was introduced by Arnett [1] to describe the period of life between the ages of 18 and 25. The most important developmental tasks during this period are reaching independence from parents, making decisions about studies and career, redefining friendships, and entering into intimate and committed romantic relationships. Emerging adulthood is an age of identity exploration and individuals usually try out different roles and possibilities. Whereas Erikson believed that the main developmental task of young adulthood was to resolve the crisis between intimacy and isolation [2], emerging adults are now considered to face several simultaneous developmental tasks as individuals are navigating multiple life domains at once. Although some tasks may take precedence at different times, they tend to interact with each other in ways that reflect the unique complexity of this life stage [1]. Emerging adulthood is characterized by more autonomy than adolescence, but less commitment than adulthood. This period is considered typical of Western industrialized societies, and the main feature is usually a prolonged exploration of different possibilities before assuming a full adult role [1,3,4]. Involvement in romantic relationships is, therefore, one of the main characteristics of emerging adulthood. Several studies have highlighted the positive role of high-quality romantic relationships in terms of identity definition and increased self-confidence as well as personal well-being in emerging adults [5]. Among the dimensions of romantic relationship quality, both intimacy and conflict play a crucial role in the development of individual romantic competence and psychological well-being.

Intimacy refers to the strength of the emotional bond between romantic partners and includes aspects such as involvement, interdependence, and attachment [6]. While the most important dimensions of a romantic relationship in adolescence are the needs for companionship, affection, and emotional support, in emerging adulthood, more intimacy and commitment represent the core dimensions of romantic involvement [7]. In addition, stronger affection, deeper intimacy, and closeness are associated with greater sexual involvement. It has been shown that intimacy with a romantic partner is generally associated with greater individual well-being, as it corresponds to the fulfillment of important developmental tasks in emerging adulthood [5,8].

Conflicts are another important dimension of romantic relationships. Disagreements and divergences between partners are likely due to different interests, needs, and sensitivities. In addition, partners usually spend much more time together in emerging adulthood than in adolescence. However, not all emerging adults have sufficiently developed their interpersonal skills, which can lead to conflictual interactions with their romantic partner [9,10]. However, conflict should not always be seen as negative for the well-being of the individual and the couple. Under certain conditions, it can be a positive factor. This depends not so much on the conflict itself, but rather on the frequency of the episodes and the methods of resolution and negotiation used, as well as the other characteristics of the relationship, including the degree of intimacy [11]. The ability to manage conflict with a romantic partner through strategies such as compromise and negotiation increases from adolescence to emerging adulthood in parallel with the development of communication and emotion regulation skills [9,12].

Thus, the quality of romantic relationships in emerging adulthood may be associated with psychological well-being or discomfort, and, in particular, depression has been considered in many studies as one of the mental health consequences associated with unsatisfying or problematic romantic relationships during this period of the life span [13]. Several studies have emphasized the role of both intimacy and conflict management in the development of depressive symptoms. On the one hand, high-quality romantic relationships characterized by intimacy and commitment were found to be protective for the development of depressive symptoms [14]. On the other hand, maladaptive conflict management strategies (e.g., attacking or denigrating the partner, showing extreme anger) are associated with the increase in depression in emerging adults, whereas constructive conflict management strategies (e.g., understanding the partner’s point of view, talking about unmet needs) are protective factors in relation to the development of depressive symptoms [11,15].

Although the link between intimacy and conflict in romantic relationships and depression has been emphasised, the role of possible mediators of this relationship remains to be explored, especially in emerging adults. Given the peculiarities of romantic relationships at this stage of the life cycle compared to adolescence and adulthood, it is important to focus attention on possible mediating variables that are particularly important for this age group. For this reason, in the present study, we focused on couple satisfaction and identity satisfaction as possible mediating factors in the relationship between romantic relationship quality (i.e., intimacy and conflict) and depressive symptoms.

Couple satisfaction refers to a subjective evaluation of the fulfillment of some key aspects of the relationship, such as desires, expectations and needs, time management and activities, and the way conflicts are addressed and resolved [16,17]. Both intimacy and conflict are predictors of couple satisfaction. Emotional intimacy implies sharing deep emotional bonds with a partner and feeling understood and cared for, which contributes to greater couple satisfaction. Similarly, constructive conflict resolution based on open communication between partners contributes to higher relationship satisfaction [18]. Despite the importance of intimacy and conflict for couple satisfaction, few studies have examined these relationships in emerging adults [19,20]. Couple satisfaction, in turn, is an indicator of a couple’s well-being and stability [17] and is associated with higher well-being and lower risk of developing depressive symptoms [14,21].

As outlined above, identity exploration is one of the characteristic features of young adulthood [1], and identity definition is involved in all developmental tasks of this age. Both autonomy and relatedness are crucial for identity development [22]. Individuals need to perceive themselves as differentiated from others, but also to feel connected and valued by them to experience both uniqueness and a sense of belonging, which are essential for healthy identity formation [23]. Specifically, the relationship with a partner provides a context for identity exploration in adulthood, as it can foster self-confidence and trust and offers the opportunity to find a balance between dependence and autonomy and to experience emotional support. In particular, some studies have emphasized the link between intimacy, alliance, and support in the couple relationship and identity development in emerging adults [24,25]. The ability of young adults to develop a coherent sense of identity across multiple domains has been shown to be associated with greater well-being and lower levels of internalizing symptoms [26,27,28].

In our study, the construct of identity was operationalized with reference to the integrative model proposed by Vignoles and colleagues [29,30] (Motivated Identity Construction Theory, MICT). According to this model, the definition of identity is guided by six psychological motives: self-esteem, efficacy, continuity, distinctiveness, belonging, and meaning. People are motivated to see themselves positively (self-esteem motive), to feel competent and able to influence their environment (efficacy motive), to maintain a sense of continuity despite significant changes in life (continuity motive), to seek a sense of differentiation from others (distinctiveness motive), to maintain a sense of closeness and acceptance by others in their social environment (belonging motive), and, finally, to find their lives meaningful (meaning motive). Greater identity satisfaction is linked to greater endorsement of all six of these motives. The model has been shown to be useful in studying emerging adult identity [31,32,33].

**Figure 1:**
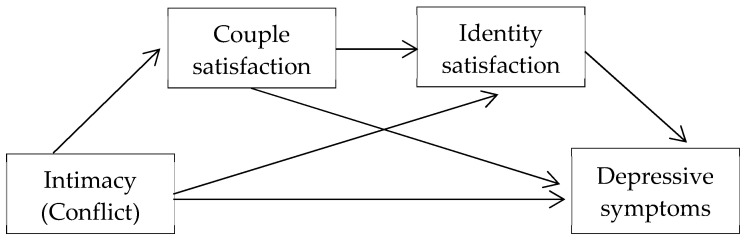
Proposed sequential mediation model.

Considering the theoretical framework outlined above, the present study tested a serial mediation model in which intimacy and conflict in romantic relationships are predictors of depressive symptoms both directly and through the mediation of couple satisfaction and identity satisfaction in a group of Italian emerging adults (Figure 1).

We formulated the following hypotheses:

**H1.**  *Greater intimacy and lower conflict in romantic relationships are directly related to fewer depressive symptoms.*

**H2.**  *Greater intimacy and lower conflict in romantic relationships are related to higher couple satisfaction and identity satisfaction, which, in turn, are related to fewer depressive symptoms.*

## 2. Materials and Methods

### 2.1. Participants

The participants were selected from a larger convenience sample of 561 emerging adults involved in a study on friendship and romantic relationships and their effect on psychological well-being. Participants were recruited on a voluntary basis from secondary school and university students in the northwest of Italy. Only people who were presently involved in a romantic relationship were selected for the present study. The final sample included 268 emerging adults (N = 161, 60% girls), aged from 18 to 23 (mean age = 19, standard deviation = 1.4). Most participants (N = 167, 62%) were high school students, 85 (32%) attended university, while the remaining 16 (6%) did not provide a response to this question. Most participants (N = 152, 57%) reported having been involved in their current romantic relationship for more than one year, 42 (16%) for a period ranging from six months to one year, and the remaining (N = 74, 27%) for less than six months. Previous studies have shown a relationship between psychological adjustment and romantic relationship quality even in short-term relationships [34]. To ensure that the sample size was sufficient, an a priori power analysis was conducted, using G*Power software 3.1.9.7. With a small to medium effect size (f^2^ = 0.15), an alpha level of 0.05, and a desired statistical power of 0.80, the required sample size was calculated to be 200 participants. The final sample of 268 participants exceeds this requirement, ensuring adequate power for the analyses.

### 2.2. Procedure and Measures

Data were collected through a self-report questionnaire, containing several questions and measures designed to investigate the characteristics of friendship and romantic relationships and several indicators of well-being/illness. The questionnaire was administered anonymously to secondary school and university students in the classrooms during class time and returned to the researchers immediately upon completion.

The study was approved by the Bioethics Committee of the University of Turin (Italy) and written informed consent was obtained from the participants before the questionnaire was administered. The participants did not receive benefits for study participation. The following questions and scales were used in the present study (the psychometric properties of which are given for the sample used in the study).

Quality of romantic relationship was evaluated through the Romance Qualities Scale (RQS) [35]. The scale is the equivalent version of the FQS (Friendship Qualities Scale) [36,37] used to investigate the qualitative aspects of romantic relationships. It comprises 22 items describing different situations typical of romantic relationships grouped into 5 dimensions (companionship, conflict, help, security, intimacy). People expressed their agreement on a 5-point Likert scale ranging from 1 (absolutely false) to 5 (absolutely true) with reference to their relationship with their current partner. For the present study, we used conflict (4 items, e.g., My partner and I can argue a lot; range 4–20; Cronbach’s alpha = 0.75; McDonald’s omega = 0.77) and intimacy (5 items, e.g., If there is something bothering me, I can tell my partner about it even if it is something I cannot tell other people; range 5–25; Cronbach’s alpha = 0.74; McDonald’s omega = 0.75).

Identity satisfaction was evaluated using the Identity Motives Scale [38], which considers the six identity motives of the Motivated Identity Construction Theory [29] (self-esteem, efficacy, continuity, belonging, distinctiveness, and meaning). It is composed of 12 items, both positive (e.g., When I think about my future, I think I will feel proud) and negative (e.g., When I think about my future, I think I will feel powerless). Each item ranges from 1 (extremely disagree) to 5 (extremely agree) and negative items are reverse coded. For the aims of the present study, the scores for the six subscales were summed up (range 12–60) and higher scores represented greater identity satisfaction. Cronbach’s alpha in our study was 0.72 and McDonald’s omega was 0.70.

Couple satisfaction was assessed through a modified version of the Couple Relationship Satisfaction Scale (CRSS) [39]. The respondents indicated how satisfied they were with diverse areas of their couple relationships (i.e., intimacy, communication, decision making, problem solving, amount of time spent together) (e.g., How satisfied are you with the way you and your partner make decisions together?). Each item was scored on a 5-point scale (ranging from 1 = very dissatisfied to 5 = very satisfied). The scale demonstrated good reliability in our study (Cronbach’s alpha = 0.81; McDonald’s omega = 0.81).

Depressive symptoms were assessed through the Center for Epidemiologic Studies Short Depression Scale (CES-D 10) [40]. It comprises 10 items investigating the presence of depressive symptoms in the previous week on a 4-point Likert response format, from 0 (rarely or none of the time) to 3 (most or all the time) (e.g., I have been concerned about things that I am not generally concerned about) (Cronbach’s alpha = 0.84; McDonald’s omega = 0.87; range 0–30; a cut off score of 10 or higher indicates the presence of significant depressive symptoms).

### 2.3. Data Analysis

Statistical analyses were performed using SPSS 29. A preliminary data check indicated that the percentage of missing responses for the study variables was <10%. The MCAR (Missing Completely at Random) test [41] was not statistically significant, indicating that missing data were completely at random. Therefore, missing data were imputed in SPSS with the EM (Expectation Maximization) procedure.

Descriptive statistics were calculated for each variable (mean, SD) as well as Pearson’s bivariate correlations among all study variables. The internal consistencies of the constructs were confirmed by Cronbach’s alpha and McDonald’s omega (Ω) coefficients [42]) and Harman’s single-factor test was used to assess common method bias [43]. The test results indicated that a single factor accounted for less than 50% of the variance, suggesting that common method bias was not a significant concern.

To test our hypothesis that couple satisfaction and identity satisfaction act as serial mediators in the relationship between intimacy/conflict and depressive symptoms, we used the SPSS PROCESS macro, Model 6 [44]. Two separate models were tested, one with intimacy, and the other with conflict as independent variables. For each model, the statistical significance of the serial mediation effect was evaluated through a bootstrapping procedure (95% confidence intervals with 5000 bootstrap samples). Confidence intervals that do not contain zero indicate a statistically significant effect.

## 3. Results

### 3.1. Descriptive Statistics

The means, standard deviations, and correlations among the study variables are reported in Table 1.

A positive correlation was found between couple satisfaction and intimacy (r = 0.50, *p* < 0.01), indicating an association between higher levels of satisfaction in the couple relationship and greater intimacy. Similarly, identity satisfaction showed a positive correlation with intimacy (r = 0.26, *p* < 0.01), suggesting that greater intimacy is associated with higher identity satisfaction. Conflict was negatively correlated with couple satisfaction (r = −0.25, *p* < 0.01) and intimacy (r = −0.21, *p* < 0.01), and positively correlated with depressive symptoms (r = 0.23, *p* < 0.01). Intimacy was negatively correlated with depressive symptoms (r = −0.31, *p* < 0.01).

### 3.2. Mediation Analyses

#### 3.2.1. Conflict as Independent Variable

Conflict was found to be a significant positive predictor of depressive symptoms, but its effect was partially mediated by satisfaction with the couple and satisfaction with identity. Specifically, higher levels of conflict were associated with lower levels of couple satisfaction, which, in turn, predicted lower levels of identity satisfaction, ultimately leading to higher depressive symptomatology.

Regarding control variables, gender had a significant effect on depressive symptoms (b = −1.97, 95% CI [−3.23, −0.71], β = −0.164), indicating that females reported higher levels of depressive symptoms compared to males.

**Figure 2:**
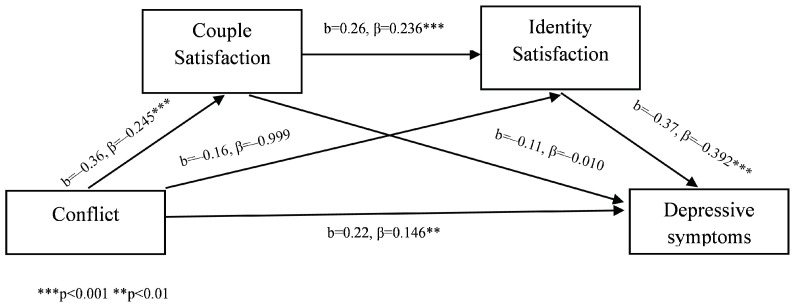
Conflict mediation model.

An examination of indirect effects showed (see Table 2) evidence of significant serial mediation effects linking conflict to depressive symptoms. The total indirect effect through couple satisfaction and identity satisfaction was significant (β = 0.13, 95% CI [0.05, 0.23]) (Figure 2).

#### 3.2.2. Intimacy as Independent Variable

Intimacy was found to be a significant negative predictor of depressive symptoms, and its effect was partially mediated by couple satisfaction and identity satisfaction. Higher levels of intimacy were associated with higher couple satisfaction, which, in turn, predicted higher intimacy and identity satisfaction, ultimately leading to fewer depressive symptoms. A statistically significant effect also emerged for the path from intimacy to depressive symptoms through identity satisfaction.

Regarding control variables, gender had a significant effect on depressive symptoms (b = −2.32, 95% CI [−3.58, −1.06], β = −0.193), indicating that females reported higher levels of depressive symptoms compared to males.

**Figure 3:**
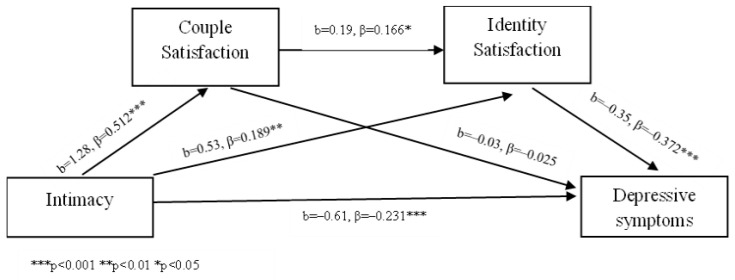
Intimacy mediation model.

An examination of indirect effects (Table 3) showed evidence of both simple and serial mediation effects linking intimacy to depressive symptoms. The total indirect effect through couple satisfaction, intimacy, and identity satisfaction was significant (β = −0.30, 95% CI [−0.51, −0.10]) (Figure 3).

## 4. Discussion

The aim of the present study was to examine the serial mediation effects of couple satisfaction and identity satisfaction in the relationship between conflict and intimacy, and depressive symptoms in young adults in romantic relationships. The results support the hypothesized model and highlight the complex pathways through which relationship dynamics influence psychological well-being.

In our study, women reported higher levels of depressive symptoms compared to men, which is consistent with the literature reporting a preponderance of depressive symptoms among women in young adulthood [45].

The results show that conflict and intimacy in romantic relationships are significant predictors of depressive symptoms, acting both directly and indirectly through couple satisfaction and identity satisfaction. Higher levels of conflict and lower levels of intimacy were associated with lower levels of couple satisfaction, which, in turn, predicted lower levels of identity satisfaction, ultimately leading to an increase in depressive symptoms. These findings underscore the critical role that relationship quality plays in the mental health of emerging adults.

Even after accounting for mediating factors, the direct effect of conflict and intimacy on depressive symptoms remained significant, suggesting that both contribute independently to depressive symptoms. This underscores the pervasive and multifaceted effects of conflict and intimacy on psychological well-being, beyond their effects on relationship satisfaction and identity.

Couple satisfaction proved to be a crucial mediator in the relationship between conflict and intimacy and depressive symptoms. This is consistent with previous research suggesting that satisfaction with one’s romantic relationship is an essential component of psychological well-being [5,8,14]. When individuals experience high levels of conflict and low intimacy, their satisfaction with the relationship decreases, which, in turn, negatively impacts their sense of identity and belonging within the relationship and ultimately leads to a deterioration in their overall mental health.

The importance of couple satisfaction as a mediator emphasises the importance of maintaining a healthy and satisfying romantic relationship [14,18]. Relationship satisfaction is often associated with numerous positive outcomes, including improved mental health, emotional well-being, and overall life satisfaction.

Similarly, identity satisfaction was found to be an important mediator in the relationship between conflict, intimacy, and depressive symptoms. In particular, the link between intimacy and depressive symptoms through the simple mediation of identity satisfaction suggests that when intimacy is reduced, the sense of identity and self-worth within the relationship may be threatened, which may then lead to increased feelings of hopelessness, worthlessness, and, ultimately, depressive symptoms. This emphasizes the fact that in emerging adulthood, the dimension of intimacy in the couple relationship plays a central role in both identity development and the containment of depressive feelings. This is consistent with the literature that emphasises the importance for young adults to invest in life projects that specifically address the affective dimension [2,46].

With regard to the specific developmental tasks of emerging adulthood, identity formation is a crucial aspect. This study shows how romantic relationships in this phase contribute significantly to identity satisfaction. Intimacy within the relationship fosters a safe environment for exploring one’s identity, while conflict, if not managed well, can disrupt this process. The findings are particularly relevant for understanding the intersection of relationship dynamics and identity satisfaction, both of which are essential developmental tasks in young adulthood.

## 5. Implications

### 5.1. Theoretical Implications

This study has theoretical implications in the following respects. First, the findings suggest the importance of considering relational and identity aspects in the psychological development of emerging adults. Studies on the role of social relationships in identity development suggest that the nature of people’s relationships has a major impact on their psychological state and well-being [46,47]. This supports the findings of this study, which demonstrate couple satisfaction and identity satisfaction as mediators of the link between conflict, intimacy, and depressive symptoms. These aspects emphasize the close interaction between relationship dimensions and identity development [46]. The quality of the social relationships that are most meaningful to the individual, especially the most intimate ones, is of central importance for identity development, especially in emerging adulthood when the main developmental task is the acquisition of an adult identity [1,48] High-quality romantic relationships during this period are also important because they are a prelude to future romantic relationships in adulthood, which are generally characterized by greater stability.

Furthermore, the results are consistent with identity theories, according to which a stable and positive identity is recommended for mental health in emerging adulthood [48]. The findings suggest that identity satisfaction is one of the mediating processes through which relationship quality influences depressive symptoms. This emphasizes that both relationship and identity aspects should be considered in various studies and in practice to promote mental health in emerging adults.

A final consideration can be made with regard to the Italian socio-cultural context in which the study was conducted. In Italy, most emerging adults still live with their parents and leave home around the age of 30, later than in other European countries [49]. Entering the labor market is often difficult and temporary, with higher unemployment rates than in other European countries, and young people generally postpone decisions for adulthood, such as entering a committed relationship and having children [50,51]. All of these factors relate to a “long transition” to adulthood among Italian emerging adults [52]. In this context, the results of our study showed the importance of a stable and satisfactory romantic relationship as an important marker of adulthood which influences both identity redefinition and emotional well-being.

### 5.2. Practical Implications

The findings of this study have important practical implications for interventions to improve the mental health of young adults in romantic relationships. Initiatives that focus on improving relationship quality, particularly by reducing conflict and increasing couple satisfaction, may be effective in mitigating depressive symptoms. Similarly, interventions that support individuals in developing a stable and positive identity in the context of their relationships may be beneficial.

Couples and relationship education programs can help couples improve their communication, strengthen their intimacy, resolve conflicts, and increase their overall relationship satisfaction. These programs can provide couples with the necessary tools and strategies to effectively manage relationship problems and, thus, reduce the negative impact of conflict on their mental health.

In addition, in some cases, individual therapy focused on identity development can help emerging adults navigate the complex process of identity formation, which is a critical developmental task at this stage.

## 6. Limitations

This study has several limitations. The cross-sectional design precludes causal inferences, so longitudinal studies are needed to establish causality and examine the temporal dynamics of the relationships between conflict, intimacy, couple satisfaction, identity satisfaction, and depressive symptoms. Considering bidirectional influences between variables, it can be assumed that higher levels of depressive symptoms might be linked to greater conflict and lower satisfaction with both relationship and identity. Future studies should utilize longitudinal methods to better understand how changes in relationship dynamics and identity satisfaction affect mental health over time.

In addition, the use of self-report measures may introduce biases, such as social desirability and recall bias. Incorporating multiple data collection methods, including observational data and partner-reported measures, would allow for a more comprehensive understanding of relationship dynamics and their effects on mental health.

The sample consisted mainly of young adults in a specific cultural context, which may limit the generalizability of the findings. Future research should include more diverse samples to increase the external validity of the results. Examining these relationships in different cultural contexts and in different populations would shed light on the extent to which the findings can be generalized.

In addition, this study focused on couple satisfaction and identity satisfaction as mediators, while other potential mechanisms, such as communication patterns, coping strategies, and social support, should be explored in future research. Understanding the full range of pathways through which relationship dynamics influence mental health will provide a more comprehensive understanding of this complex topic.

## 7. Conclusions

In summary, this study underscores the considerable influence of conflict and intimacy on depressive symptoms in emerging adults in romantic relationships, with couple satisfaction and identity satisfaction serving as important mediating factors. The findings emphasize the importance of addressing relationship quality and identity-related issues in interventions to improve mental health in this population. By cultivating healthy relationship dynamics and promoting positive identity development, it may be possible to reduce the prevalence and severity of depressive symptoms in young adults.

This study contributes to the existing literature by highlighting the complex pathways through which relationship conflict and intimacy impact mental health and by emphasizing the intertwined nature of relationship and identity-related factors in emerging adulthood. The findings are important for both theoretical understanding and practical application, and provide a foundation for future research and interventions to promote the overall well-being of young adults in romantic relationships.

## Figures and Tables

**Table 1:** Bivariate correlations between study variables (N = 268).

	Variables	M (SD) or %	1	2	3	4	5	6
1	Gender	F = 60.07%	—					
2	Age	19.0 (1.4)	0.03	—				
3	Couple satisfaction	41.26 (5.61)	−0.01	0.05	—			
4	Depressive symptoms	8.94 (5.91)	−0.19 **	0.02	−0.24 **	—		
5	Identity satisfaction	45.01 (6.33)	0.08	0.01	−0.26 **	−0.45 **	—	
6	Conflict	13.15 (3.79)	0.03	−0.07	−0.25 **	0.23 **	−0.15 **	—
7	Intimacy	23.49 (2.25)	−0.14 *	0.03	0.50 **	−0.31 **	0.26 **	−0.21 **

Note. Gender (1 = male, 0 = female); * *p* < 0.05, ** *p* < 0.01.

**Table 2:** Estimated indirect effects and 95% confidence intervals (5000 bootstrap samples) for conflict.

		95% Confidence Interval		
Route of Indirect Effect	Effect	Lower Limit	Upper Limit	Standardized Effect
Conflict → Couple Satisfaction → Depressive Symptoms	0.0382	−0.0153	0.0998	0.0245
Conflict → Identity Satisfaction → Depressive Symptoms	0.0606	−0.0163	0.1439	0.0388
Conflict → Couple Satisfaction → Identity Satisfaction → Depressive Symptoms	0.0354	0.0130	0.0667	0.0227
Total Indirect Effect	0.1342	0.0459	0.2299	0.0861

**Table 3:** Estimated indirect effects and 95% confidence intervals (5000 bootstrap samples) for intimacy.

		95% Confidence Interval		
Route of Indirect Effect	Effect	Lower Limit	Upper Limit	Standardized Effect
Intimacy → Couple Satisfaction → Depressive Symptoms	−0.0335	−0.2301	0.1663	−0.0127
Intimacy → Identity Satisfaction → Depressive Symptoms	−0.1848	−0.3270	−0.0450	−0.0703
Intimacy → Couple Satisfaction → Identity Satisfaction → Depressive Symptoms	−0.0831	−0.1790	−0.0102	−0.0316
Total Indirect Effect	−0.3014	−0.5052	−0.0968	−0.1146

## Data Availability

The data presented in this study are available on request from the corresponding author due to privacy reasons.
